# Pros and cons of insulin administration on liver glucose metabolism in strength-trained healthy mice

**DOI:** 10.1590/1414-431X20187637

**Published:** 2019-01-24

**Authors:** V.A.R. Pereira, K.S. Vedovelli, G.Y. Muller, Y.F. Depieri, D.H.C.G. Avelar, A.H.E. de Amo, D.R. Jimenes, J.N.L. Martins, A.C. Silvério, C.R.G. Gomes, V.A.F. Godoi, M.M.D. Pedrosa

**Affiliations:** 1 Programa de Pós-Graduação em Ciências Fisiológicas, Universidade Estadual de Maringá, Maringá, PR, Brasil; 2 Especialização em Fisiologia Humana, Departamento de Ciências Fisiológicas, Universidade Estadual de Maringá, Maringá, PR, Brasil; 3 Graduação em Educação Física, Universidade Estadual de Maringá, Maringá, PR, Brasil; 4 Graduação em Medicina, Universidade Estadual de Maringá, Maringá, PR, Brasil; 5 Graduação em Educação Física, Centro Metropolitano de Maringá, Maringá, PR, Brasil; 6 Graduação em Ciências Biológicas, Universidade Estadual de Maringá, Maringá, PR, Brasil; 7 Especialização em Anatomia e Fisiologia, Departamento de Ciências Morfológicas, Universidade Estadual de Maringá, Maringá, PR, Brasil; 8 Programa de Pós-Graduação em Biologia Celular e Molecular, Universidade Estadual de Maringá, Maringá, PR, Brasil; 9 Graduação em Biotecnologia, Universidade Estadual de Maringá, Maringá, PR, Brasil; 10 Departamento de Ciências Morfológicas, Universidade Estadual de Maringá, Maringá, PR, Brasil; 11 Departamento de Ciências Fisiológicas, Universidade Estadual de Maringá, Maringá, PR, Brasil

**Keywords:** Muscle strength, Gluconeogenesis, Liver, Adiposity, Training

## Abstract

Non-diabetic individuals use hormones like insulin to improve muscle strength and performance. However, as insulin also leads the liver and the adipose tissue to an anabolic state, the purpose of this study was to investigate the effects of insulin on liver metabolism in trained non-diabetic Swiss mice. The mice were divided into four groups: sedentary treated with saline (SS) or insulin (SI) and trained treated with saline (TS) or insulin (TI). Training was made in a vertical stair, at 90% of the maximum load, three times per week. Insulin (0.3 U/kg body weight) or saline were given intraperitoneally five times per week. After eight weeks, tissue and blood were collected and *in situ* liver perfusion with glycerol+lactate or alanine+glutamine (4 mM each) was carried out. The trained animals increased their muscle strength (+100%) and decreased body weight gain (–11%), subcutaneous fat (–42%), mesenteric fat (–45%), and peritoneal adipocyte size (–33%) compared with the sedentary groups. Insulin prevented the adipose effects of training (TI). The gastrocnemius muscle had greater density of muscle fibers (+60%) and less connective tissue in the trained groups. Liver glycogen was increased by insulin (SI +40% and TI +117%), as well as liver basal glucose release (TI +40%). Lactate and pyruvate release were reduced to a half by training. The greater gluconeogenesis from alanine+glutamine induced by training (TS +50%) was reversed by insulin (TI). Insulin administration had no additional effect on muscle strength and reversed some of the lipolytic and gluconeogenic effects of the resistance training. Therefore, insulin administration does not complement training in improving liver glucose metabolism.

## Introduction

A rush of information from the media is persuading people to seek methods often questionable relative to health and legal principles, aiming at obtaining a physical appearance socially regarded as ideal. Among these methods, it is known that the indiscriminate use of hormones to optimize the results of training is becoming frequent among bodybuilders (1–3). One of these hormones is insulin (4,5). Insulin can be easily purchased in drugstores without prescription and is relatively inexpensive (especially regular insulin).

Insulin acts in synergy with growth hormone (GH) to promote muscle hypertrophy; GH stimulates the production of insulin-like growth factors, which in turn directly stimulate protein synthesis and increase skeletal muscle insulin sensitivity (6–8). On the other hand, insulin stimulates lipid synthesis (9), thereby increasing adipose tissue and decreasing performance in most high-performance sports and many types of training. In addition, for bodybuilders and practitioners of fitness activities in general, this can lead to undesirable percentages of fat mass. Insulin is also an important signaling agent in the liver, where it regulates energy metabolism by promoting *de novo* lipogenesis and glycogen synthesis (10). Therefore, insulin administration may have several acute and chronic outcomes for trainers, which are seen even in type 1 diabetic patients, obligatory users of this hormone (11).

Studies assessing the adaptive responses to aerobic exercise are found in the literature. They have shown that aerobic exercise is efficient in decreasing serum triacylglycerols (TAGs) and free fatty acids (FFAs), diminishing markers of lipid synthesis and liver lipid content, as well as increasing mitochondrial oxidation and liver activity of phosphoenolpyruvate carboxy kinase (PEPCK), a regulatory enzyme of gluconeogenesis (12–14). A study by Hallsworth et al. (15), using resistance training in patients with non-alcoholic fatty liver disease, showed that after eight weeks of training the patients had decreased intra-hepatic lipid content, improved lipid oxidation, and decreased insulin resistance.

Controlled studies on the use and action of insulin associated with exercise in non-diabetic animal models, in which it is possible to accurately assess the effects of these interventions, are rare (16,17). Increases in protein and DNA content and rate of protein synthesis of soleus muscle of strength-trained rats were reported (17), but changes in muscle protein and liver and muscle glycogen were not found in another investigation (16).

As insulin is a hormone with systemic metabolic actions, it was hypothesized that its non-clinical use aimed at improving muscle strength could change liver glucose metabolism. Therefore, the purpose of this work was to test the effects of resistance training and the possible influence of low doses of insulin on liver gluconeogenesis and glucose homeostasis in healthy Swiss mice. In addition, some histological aspects of skeletal muscle and adipose tissue were assessed. Strength increased significantly after eight weeks of training, and this was accompanied by lower adiposity, plasma cholesterol and liver glycogen content, higher muscle fiber density, and increased liver gluconeogenesis from amino acids. Except for fiber density and maximum load, these effects were reversed by insulin administration to trained mice.

## Material and Methods

**Figure 1.**
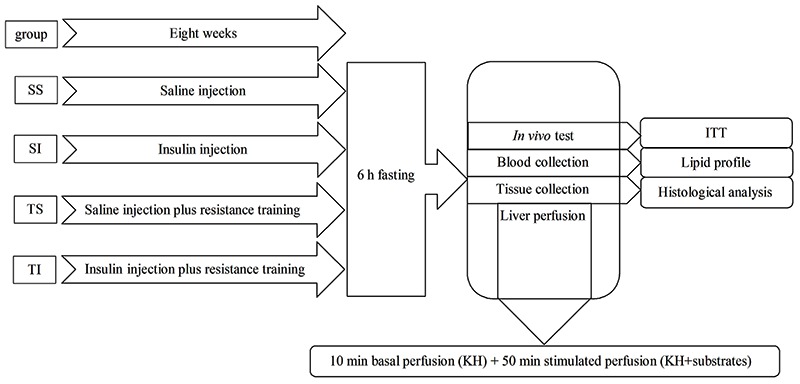
Schematic representation of the experimental protocols of Swiss mice of groups SS: sedentary treated with saline; SI: sedentary treated with insulin; TS: trained treated with saline; and TI: trained treated with insulin. ITT: insulin tolerance test; KH: Krebs-Henseleit.

This investigation used 120 Swiss mice aged 40 days and weighing 30–35 g at the beginning of the protocols. They were given free access to water and rodent chow and were kept at constant light/dark cycles (12 h light/12 h dark) and temperature (22±2°C). The experimental protocols were approved by the Ethics Commission on the Use of Animals of the State University of Maringá (certificate 8548200616/2016). The experimental design is shown in Figure 1.

### Experimental groups

The animals were divided into four experimental groups: sedentary treated with saline (SS, n=30), sedentary treated with insulin (SI, n=30), trained treated with saline (TS, n=30), and trained treated with insulin (TI, n=30). Regular insulin, at the dose of 0.3 U/kg body weight (b.w.) (17), was dissolved in saline (3 mL/kg b.w.; Novolin^®^, Novo Nordisk, Brazil) and given intraperitoneally, once a day (at 2 pm), five days per week for eight weeks, to the mice from groups SI and TI. The same volume of saline was injected in the animals from groups SS and TS.

### Maximum load test and training protocol

The resistance training apparatus was adapted for mice from previous studies (18–20). It consisted in a stair measuring 105 cm long, 8 cm wide, and inclined 80°. At the top, there was a 12-cm^3^ chamber for the animal to rest between series. Each series corresponded to a complete climbing. The maximum load (ML) tests and the training sessions were carried out in the morning (8:00–10:00 am). The load was put into a tube attached to the tail of the animal.

The first ML test started with a load corresponding to 75% b.w. A load of 8 g was added after each series. A one-min interval was allowed between series. The ML was established as the load of the last complete climbing. Each week a new ML test was performed, but starting from the ML of the previous week, to adjust the training intensity (19,20). Mice from groups SS and SI were subjected to the ML test on the first and last week of the experimental period.

The training sessions were carried out three times per week on alternate days for eight weeks. They were carried out with 90% of the ML and were composed of three to nine complete series. Exhaustion was considered as the moment when the mouse could no longer reach the top of the stair. When exhaustion did not take place, training was finished after nine series. No painful stimuli were applied to make the mice continue the training session. A one-min interval was allowed between series (20). Training was not performed on the day of the experiments.

### Insulin tolerance test

At the end of eight weeks, some of the mice of each group were subjected to the insulin tolerance test (ITT) after a six-hour fast. They were given 1 U/kg b.w. of regular insulin *ip* (Novolin^®^, 5 mL/kg b.w.) and blood glucose was assessed with test strips and glucometer (AccuCheck^®^, Abbott, Brazil) at times 0, 5, 10, 15, 20, 25, 30, and 60 min; 0 min being the moment just prior to insulin injection. The glucose decay index (kITT) was calculated for the first 30 min of the test (21).

### Tissue and blood collection

Forty-eight hours after the ITT, the animals were again fasted for six hours and euthanasia was performed (thionembutal 120 mg/kg b.w. after lidocaine 5 mg/kg b.w., *ip*). White adipose tissue (subcutaneous, peritoneal, mesenteric, and epididymal), skeletal muscle (triceps and gastrocnemius), and liver were removed and weighed. Blood was collected and centrifuged at 1,600 *g* for 5 min at room temperature, and the plasma was stored for further biochemical analyses. Blood glucose was determined by test strips and glucometer (AccuCheck^®^).

### Histological analysis of muscle and liver

Muscle and liver samples were fixed in Bouin and alcoholic formalin, respectively, for 24 h, dehydrated in ascending series of ethanol, diaphanized in xylene, included in paraffin, and sectioned in a microtome (Leica^®^ RM2245; Leica Microsystems, Germany) at 6-µm thickness. The histological sections of muscle were stained with hematoxylin-eosin. Liver sections were stained with Schiff's periodic acid (PAS) to stain glycogen deposits.

The slides were analyzed in an optical microscope (Olympus^®^ BX 50; Japan) coupled to an image analyzer (Image ProPlus^®^ 4.0; Media Cybernetics, USA). Random non-sequential digital images were captured under 20× objective for muscle and 40× objective for liver. The diameter of 120 muscle fibers of each group and the glycogen area of 120 hepatocytes of each group were determined with the image analyzer. For muscle fiber density, each microscopic field was divided in four quadrants of 232×174 pixels each, always centered at the same point in every image. One quadrant was chosen from each image (10 images/group) and its cells were counted. The choice was based on the following criteria: lesser amount of connective tissue and greater integrity of the muscle fibers.

### Histological analysis of white adipose tissue

The peritoneal fat was fixed in 4% paraformaldehyde for eight hours, then washed and kept in 70% ethylic alcohol. The tissue was dehydrated in ascending series of alcohol, diaphanized in xylene, included in paraffin, sectioned at 6-μm thickness, and stained with hematoxylin-eosin. The images were captured in an optical microscope (Nikon Eclipse E110^®^, Japan) under 20× objective. The cell diameter of 12 adipocytes from 10 images of each group were randomly measured with image analyzer Image-Pro Plus^®^.

### *In situ* liver perfusion

For this set of experiments, the 6-h fasted mice were anesthetized with thionembutal (40 mg/kg b.w. after lidocaine 5 mg/kg b.w., *ip*) and subjected to laparotomy and cannulation of the portal vein and inferior cava vein below the liver. The organ was perfused with Krebs-Henseleit (KH) buffer (pH 7.4, aerated with 95% O_2_/5% CO_2_, and warmed at 37°C) in a non-recirculating system. The perfusion fluid entered the liver through the portal vein and left through the inferior cava vein. Immediately after cannulation the diaphragm was sectioned for euthanasia. After a 30-min stabilization, samples of the effluent fluid were collected through the inferior cava vein every five min. During the period of collection, the liver was perfused for 10 min with KH alone (basal perfusion) and then for 50 min with KH containing gluconeogenic precursors (lactate+glycerol or alanine+glutamine, 4 mM each); this was the stimulated perfusion.

### Biochemical analyses

Plasma samples were used to determine the content of FFAs (Wako Chemicals; USA), glucose, total cholesterol, high-density lipoprotein (HDL) cholesterol, and TAGs (Gold Analisa^®^, Brazil) according to the specifications of the suppliers. The values of LDL and very low-density lipoprotein (VLDL) cholesterol were obtained from Friedewald's formula. The samples of the effluent fluid of the perfusion were used to determine glucose and total nitrogen by enzymatic-colorimetric methods (Gold Analisa^®^) and lactate and pyruvate through enzymatic methods. Plasma insulin was determined through radioimmunoassay (22).

### Statistical analysis

Data are reported as means±SE and were subjected to Shapiro-Wilk and Kolgomorov-Smirnof normality tests. The experimental groups were compared by one-way ANOVA-Tukey for parametric data sets. The significance level was 5%. Statistical analysis and graphs were made in Prism^®^ 5.0 (GraphPad, USA).

## Results

During the first week of training, all the mice from groups TS and TI completed the nine series of the session. As the weeks went on, the maximum load progressively increased and the number of series per training session decreased. After eight weeks (that is, at the end of the training period), most of the animals could complete only three series during each session.

**Figure 2.**
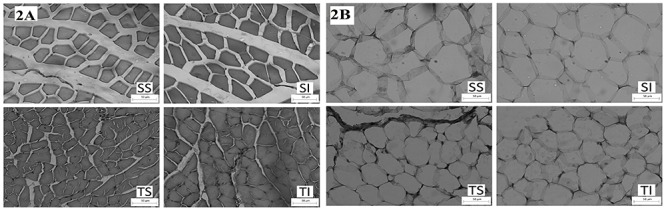
Representative histological sections of gastrocnemius muscle (**A**) and peritoneal adipose tissue (**B**) of Swiss mice of groups SS: sedentary treated with saline; SI: sedentary treated with insulin; TS: trained treated with saline; and TI: trained treated with insulin. Hematoxylin-eosin staining.

The quantitative data is shown in Table 1. The animals of groups TS and TI increased their muscle strength more than 100% compared with the sedentary groups, as assessed by the relative ML at the end of the training period of eight weeks. The relative muscle weight did not reflect this increased strength in the trained animals, as no significant difference was observed in the weight of the triceps and gastrocnemius (P>0.05). Histological analysis of the gastrocnemius did not reveal a significant difference in the mean diameter of the muscle fibers among the groups (P>0.05), but the density of muscle fibers was higher in the trained animals, while the interstitial tissue was decreased (Figure 2A). The increased muscle strength was accompanied by lower final body weight in the trained groups: on average, the sedentary mice increased their body weight by 36% during the eight-week period, while the weight gain for trained animals treated with saline (TS) was 28% and for those treated with insulin (TI) was 17%. As a result, final body weight was significantly lower in the trained groups compared with their sedentary counterparts.

**Table 1.** Quantitative data of Swiss mice of groups SS: sedentary treated with saline; SI: sedentary treated with insulin; TS: trained treated with saline; and TI: trained treated with insulin.

	SS	SI	TS	TI
Body measures (n=20–30)				
Initial body weight (g)	32.98±0.91	33.93±0.75	32.31±0.36	34.52±0.52
Final body weight (g)	44.84±0.75	46.34±0.81	41.04±0.74^ab^	39.73±0.96^ab^
Naso-anal length (cm)	10.76±0.08	10.63±0.16	10.47±0.12	10.36±0.26
Maximum load (n=20–30)				
Initial (g/10 g)	11.35±0.50	12.94±0.72	12.87±0.61	12.67±0.73
Final (g/10 g)	13.32±0.52	13.23±0.80	26.43±0.89^ab^	28.31±1.13^ab^
Adipose tissue (n=5–6)				
Subcutaneous weight (g/10 g)	0.087±0.009	0.079±0.003	0.050±0.004^ab^	0.078±0.009^c^
Mesenteric weight (g/10 g)	0.162±0.010	0.148±0.015	0.090±0.006^a^	0.122±0.011
Epididymal weight (g/10 g)	0.146±0.011	0.132±0.016	0.136±0.013	0.158±0.014
Peritoneal weight (g/10 g)	0.047±0.005	0.042±0.007	0.029±0.005	0.048±0.009
Visceral weight (g/10 g)	0.355±0.025	0.322±0.033	0.266±0.027	0.328±0.027
Peritoneal cell diameter (µm)	60.12±0.997	59.84±2.789	40.57±2.638^ab^	49.94±1.433
Skeletal muscle (n=5–6)				
Triceps weight (g/10 g)	0.058±0.005	0.060±0.002	0.056±0.007	0.070±0.002
Gastrocnemius weight (g/10 g)	0.086±0.002	0.098±0.003	0.092±0.004	0.095±0.002
Gastrocnemius fiber diameter (µm)	32.92±0.797	33.59±0.672	32.22±0.744	31.08±1.409
Gastroc. fiber density (cells/quadrant)	12.87±0.789	13.26±0.459	20.75±1.368^ab^	22.02±1.018^ab^
Liver (n=5–6)				
Liver weight (g/10 g)	0.512±0.016	0.535±0.015	0.527±0.008	0.505±0.016
Glycogen area (µm^2^)	0.103±0.008	0.142±0.004	0.080±0.010^b^	0.174±0.002^ac^

Data are reported as means±SE. ^a^P<0.05 *vs* SS; ^b^P<0.05 *vs* SI; ^c^P<0.05 *vs* TS (ANOVA-Tukey). Gastroc.: gastrocnemius; visceral: sum of mesenteric, epididymal, and peritoneal adipose tissues.

Table 1 also shows other biometric data after eight weeks of resistance training. Subcutaneous fat was decreased by training (TS), an effect not observed when training and insulin were associated (TI, P>0.05 compared with SS and SI). Mesenteric fat weight was lower in group TS compared to SS, and again insulin administration to the trained animals (TI) resulted in mesenteric fat weight not different from sedentary animals (P>0.05).

The other fat tissues had only a tendency to lower values in group TS (P>0.05) compared with the others. For instance, peritoneal fat weight of group TS was 38% lower compared with group SS. Accordingly, visceral fat weight (the sum of mesenteric, epididymal, and peritoneal fats) of the trained group treated with saline (TS) was 25% lower than in the other groups. These differences, however, did not reach statistical significance. On the other hand, the mean diameter of the peritoneal adipocytes in group TS was lower (–33%) than in the sedentary groups, and marginally lower (–20%, P>0.05) than in the trained group treated with insulin (Table 1, Figure 2B).

The relative weight of the liver was similar across the groups (P>0.05, Table 1). The analysis of liver glycogen indicated larger deposits in the groups treated with insulin (SI and TI) than in those treated with saline (SS and TS), a difference that was significant in group TI against groups SS and TS. Group TS had the smallest glycogen content. In all the groups, the liver did not show evidence of altered lobular morphology, sinusoidal organization, or of lipid droplets.

Table 2 shows the plasma profile of the groups. The animals of group TS had lower levels of total cholesterol compared with the other groups (significant compared with group SS). The levels of HDL, LDL, and VLDL cholesterol did not differ significantly (P>0.05) among the groups, as well as the plasma levels of FFAs, TAGs, fasting blood glucose, and insulin (Table 1).

**Table 2.** Plasmatic parameters of Swiss mice of groups SS: sedentary treated with saline; SI: sedentary treated with insulin; TS: trained treated with saline; and TI: trained treated with insulin.

	SS	SI	TS	TI
FFA (mEq/L)	0.092±0.009	0.085±0.010	0.080±0.010	0.076±0.005
TAG (mg/dL)	60.80±2.89	68.50±4.17	58.50±6.10	47.75±7.50
Total Chol. (mg/dL)	147.40±7.87	135.50±20.87	115.70±4.68^a^	122.30±2.09
HDL (mg/dL)	55.00±9.17	50.63±7.66	47.40±4.84	36.67±2.03
LDL (mg/dL)	76.88±5.04	65.55±4.62	56.45±7.57	73.28±2.44
VLDL (mg/dL)	13.55±1.51	14.82±1.49	10.50±1.56	12.34±2.94
Glucose (mg/dL)	127.8±14.39	153.5±14.57	150.5±10.41	160.4±18.40
Insulin (ng/mL)	0.225±0.076	0.274±0.031	0.307±0.620	0.181±0.049

Data are reported as means±SE for n=5-8/group. ^a^P<0.05 *vs* SS (ANOVA-Tukey).

FFA: free fatty acid; TAG: triacylglycerol; Chol.: cholesterol; HDL: high-density lipoprotein; LDL: low-density lipoprotein VLDL: very low-density lipoprotein.

**Figure 3.**
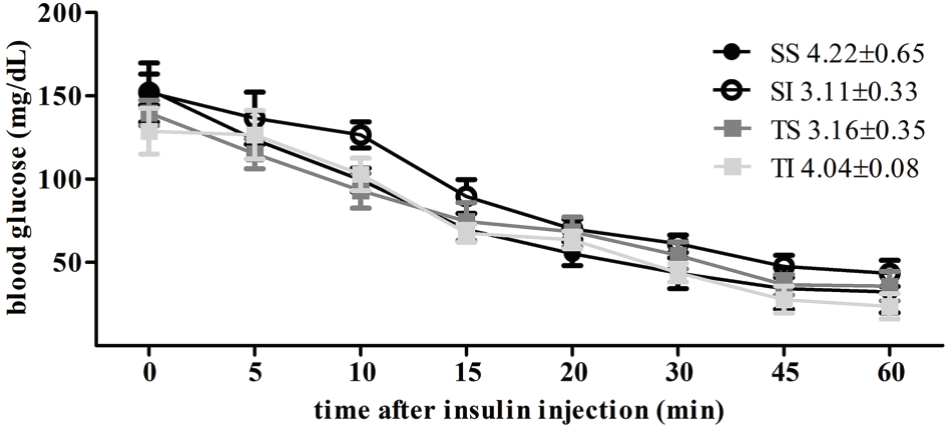
Blood glucose profile during insulin tolerance test and rate of blood glucose decay (kITT, numerical values) of Swiss mice of groups SS: sedentary treated with saline; SI: sedentary treated with insulin; TS: trained treated with saline; and TI: trained treated with insulin. Data are reported as means±SE for n=5–7/group.

The results of the ITT are shown in Figure 3. Blood glucose at the end of the test (60 min) was about 2/3 of the initial value (time 0 min) in all the groups. The statistical comparison at each time-point did not show significant differences among the groups (P>0.05). The index of blood glucose decay (kITT, numerical data in Figure 3) was not significantly changed (P>0.05) by training and/or insulin administration.

Basal liver glucose release, that is, during the first 10 min of the perfusion and without the addition of gluconeogenic precursors, was as follows (in µmol/g liver): SS, 1.259±0.121 (n=11); SI, 1.225±0.067 (n=12); TS, 1.465±0.117 (n=12); and TI, 1.743±0.140 (n=8). This last value was significantly higher (P<0.05) than in the sedentary groups.

**Figure 4.**
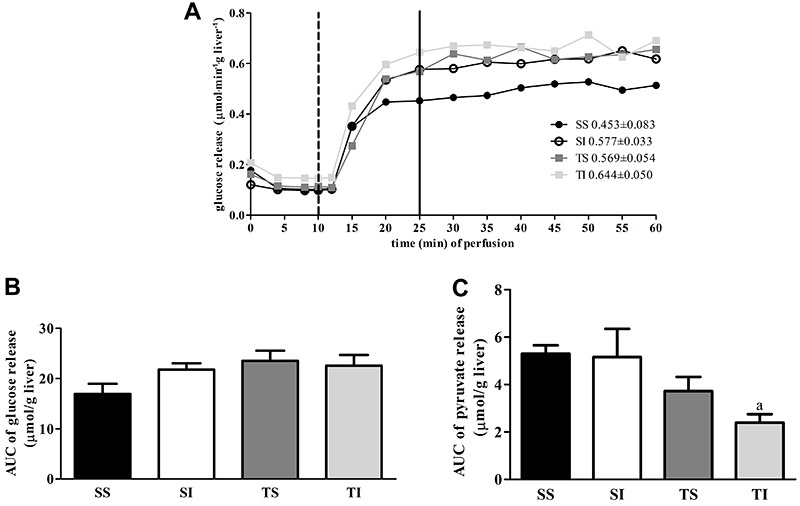
Kinetics and steady-state (**A**, numerical values) of liver glucose release and area under curve (AUC) of glucose (**B**) and pyruvate (**C**) release during perfusion with lactate+glycerol of Swiss mice of groups SS: sedentary treated with saline; SI: sedentary treated with insulin; TS: trained treated with saline; and TI: trained treated with insulin. Data are reported as means±SE for n=5–7/group.^a^P<0.05 *vs* SS (ANOVA-Tukey).

**Figure 5.**
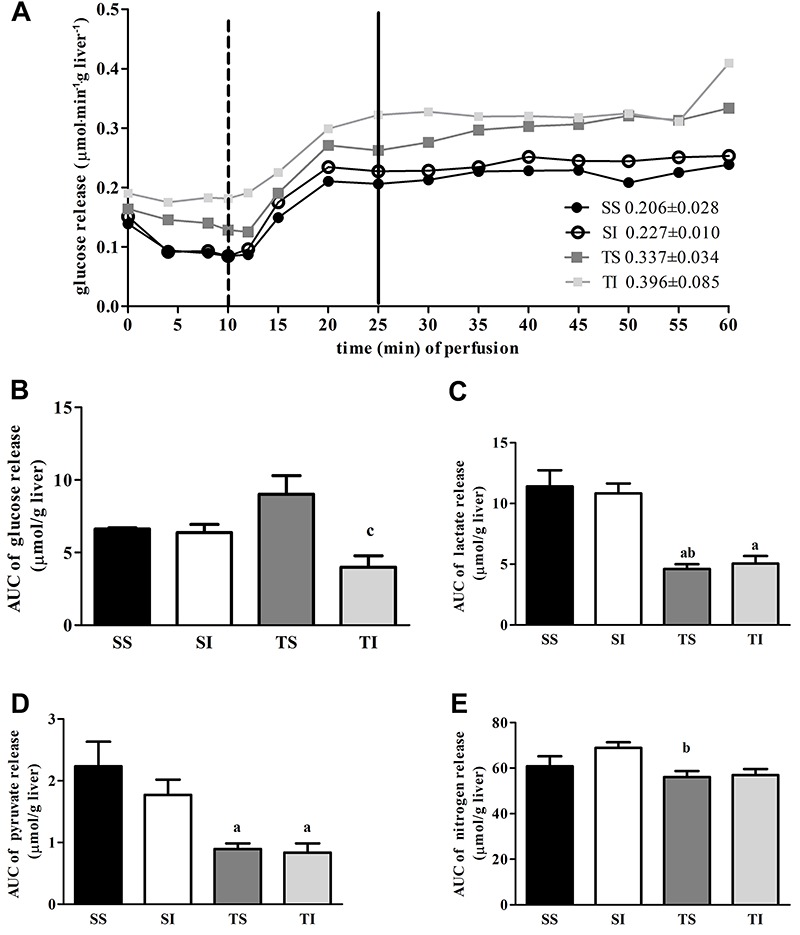
Kinetics and steady-state (**A**, numerical values) of liver glucose release and area under curve (AUC) of glucose (**B**), lactate (**C**), pyruvate (**D**), and nitrogen (**E**) release during perfusion with alanine+glutamine of Swiss mice of groups SS: sedentary treated with saline; SI: sedentary treated with insulin; TS: trained treated with saline; and TI: trained treated with insulin. Data are reported as means±SE for n=5-7/group.^a^P<0.05 *vs* SS; ^b^P<0.05 *vs* SI; ^c^P<0.05 *vs* TS (ANOVA-Tukey).

The steady-state of glucose release in the presence of gluconeogenic precursors (lactate+glycerol or alanine+glutamine) was set as the first time-point when glucose release was not different from the previous and the next time-point in group SS. This was reached at time 25 min (that is, 15 min after the infusion of the gluconeogenic precursors (continuous vertical line in Figures 4A and 5A), and hence glucose release at this point was compared among the groups; no significant differences were observed (P>0.05).

Liver glucose release in the presence of lactate+glycerol was similar in all groups (P>0.05, Figure 4B), but pyruvate release was reduced in the trained groups, being significantly lower in group TI compared with group SS (Figure 4C).

In the presence of alanine+glutamine, liver glucose release tended to be 50% higher in group TS compared with the sedentary groups (P>0.05, Figure 5B), but only the lower glucose release of group TI was significantly different from that of TS: in relative terms, glucose release was 2.2 times higher in group TS than in group TI.

Liver lactate and pyruvate release in the presence of alanine+glutamine was decreased by about 50% in the trained groups (Figure 5C and D, respectively). Nitrogen (ammonia+urea) release was lower in group TS than in group SS (Figure 5E).

## Discussion

Insulin administration alone (group SI) did not change any of the biometric, plasmatic, or metabolic parameters analyzed compared with sedentary mice treated with saline (group SS); in other words, the low doses of insulin, *per se*, did not compromise the general health status of the animals.

However, it is relevant to note that mortality was higher in group TI (=4) than in the other groups (SS=0; SI=1; TS=1), suggesting a dangerous interaction between training and insulin, albeit care was taken not to train the animals during peak insulin activity. The latter took place during the dark period of the cycle, when the animals were feeding in their cages, while training was carried out in the morning in the immediate post-prandial state. Exercise increases early and late glucose uptake by skeletal muscle, as well as insulin sensitivity in muscle (23,24) and liver (25). Although signs of convulsion or bleeding were not detected in the dead mice of group TI, the possibility of death by hypoglycemia cannot be discarded. Recurrent hypoglycemic episodes lead to unawareness and delay the trigger of hormonal and neural counter-regulatory responses until progressively lower blood glucose levels are reached, which in turn can make blood glucose recovery unattainable (11,12).

Resistance training is known to increase muscle strength and stimulate muscle hypertrophy; therefore, the increased strength observed here, even not accompanied by increased muscle weight – in this study represented by triceps and gastrocnemius – at the end of eight weeks of training was in accordance with the literature (18). Interestingly, insulin, despite being an important anabolic hormone, did not cause any additive effect on muscle strength or weight, contrary to the expected and popularly proclaimed (4,26). Also, the diameter of the muscle fibers was not changed. The higher density of muscle fibers and reduced connective tissue seen in the histological sections in the trained groups was not influenced by insulin, but only by training. Unlike other training protocols in stair for eight weeks (18,27,28), the protocol using 90% ML was shown to be efficient in reducing body weight, while it markedly increased muscle strength, as assessed by the ML at the end of the training period.

The decreased body weight was also correlated with a decreased weight of the adipose tissue in group TS. The effects of training on adipose tissue, especially the subcutaneous fat, is well established (29). In the peritoneal fat, the decreased weight in group TS was not statistically significant, but the adipocytes were smaller than in the other groups. Training decreases adipocyte size and lipid content, directly because of the energy demand (30) and the release of myokines, which induce the expression of markers of the more oxidative beige adipose tissue (31,32). It was noticed as well that insulin prevented the effects of resistance training on adipose tissue, especially in terms of fat weight. The anabolic nature of insulin manifests itself in the adipose tissue by stimulation of the uptake of fatty acids (through lipoprotein lipase) and glucose (through GLUT4), expression and activity of lipogenic enzymes (acetyl-CoA carboxylase, fatty acid synthase) and glyceraldehyde-3-phosphate dehydrogenase, and of protein FSP27, which is responsible for the formation of unilocular vesicles in adipocytes. Insulin also inhibits lipolysis in white adipose tissue (33).

The levels of FFAs and TAGs were kept within normal ranges in all the groups compared with the control (SS), in contrast to some studies pointing to a diminishment of these levels because of resistance training (34). It should be stressed, though, that the mice in this study were not obese, diabetic, or dyslipidemic, conditions in which resistance training could have a greater impact on the lipid profile.

Surprisingly, even the animals being fasted for six hours, group TI had liver basal glucose release (that is, before the infusion of gluconeogenic precursors) higher than the other groups. This release certainly comes from endogenous sources, that is, from glucose previously present in the liver. Insulin administration could have increased glycogen synthesis in the hepatocytes, given its reciprocal regulation of key enzymes such as glycogen synthase and phosphorylase (10). This possibility is reinforced by the larger glycogen content found in the trained group treated with insulin. Despite this increased basal glucose release, this group (TI), as well as the others, did not show significant alterations in fasting blood glucose, plasma insulin, or insulin sensitivity when the hormone was administered as a *bolus* (ITT), indicating that none of these interventions (insulin and training), combined or isolated, interfered with whole-body glucose homeostasis.

As one of the primary purposes of this investigation was to know whether insulin administration and resistance training could influence the liver gluconeogenic metabolism, two liver perfusion protocols were conducted: 1) using two non-proteic gluconeogenic precursors – lactate and glycerol – and 2) using two amino acids – alanine and glutamine. Alanine enters the gluconeogenic pathway through transamination with alfa-ketoglutarate catalyzed by alanine amino transferase, yielding pyruvate and glutamate, respectively. Pyruvate then proceeds to phosphoenolpyruvate by the enzymes pyruvate carboxylase and PEPCK (10,35). Glutamine is abundantly synthesized in skeletal muscle (36). In the liver, it is metabolized by glutaminase, resulting in ammonia and glutamate. The glutamate pool in the liver can be processed by glutamate dehydrogenase and originate more ammonia and carbon skeletons, which can be used as metabolic fuel, for gluconeogenesis or amino acid synthesis (36). The ammonia released by amino acid breakdown is in large part converted to urea. Lactate is oxidized to pyruvate by lactate dehydrogenase (LDH). In the gluconeogenic pathway, therefore, these three substrates – lactate, alanine, and glutamine – are channeled to phosphoenolpyruvate through pathways that include mitochondrial steps. Glycerol enters gluconeogenesis as dihydroxyacetone-phosphate and is the only one metabolized exclusively by cytosolic steps (10,25).

In the first protocol, the decreased release of pyruvate in group TS was not significant, but it was marked in the trained animals that were given insulin (TI). As pyruvate (in this protocol derived from the infused lactate) is substrate for several metabolic processes, the interpretation of this result by the methodology used is not conclusive, yet it subsidizes speculations. It is possible that the combination of insulin and training could have an inhibitory effect, either direct or indirect (for instance, reduction of the cytosolic pool of NAD^+^), on LDH, thereby decreasing the formation of cytosolic pyruvate. Alternatively, this combination could stimulate mitochondrial respiration and pyruvate oxidation by the citric acid cycle. In this case, the maintenance of the liver glucose release in group TI would be due to glycerol. On the other hand, as the pyruvate released during liver perfusion is surplus, that is, not used by the liver, and as liver glucose release was not changed by insulin combined with training, it could be that insulin-activated pathways, such as glycerol-dependent esterification of FFAs, would be consuming glycerol. This would decrease the availability of glycerol, increase the shift of pyruvate to gluconeogenesis, and decrease the release of pyruvate by the liver. In this case, the maintenance of the liver glucose release would be due to pyruvate.

The higher liver glucose release in the presence of alanine+glutamine (second perfusion protocol) of the trained animals that were given saline (TS) was accompanied by reduced release of pyruvate, lactate, and nitrogen. It is possible that all the mitochondrial steps of gluconeogenesis had been accelerated by training, hence draining lactate and pyruvate for glucose production. The decreased nitrogen release in this group could be linked to a greater capacity of nitrogen recycling in biosynthetic and/or nitrogen-saving pathways, typical of periods of high protein turnover. In this case, transamination pathways can be more active and also account for the decreased release of pyruvate and lactate. However, the decreased release of nitrogen was small compared to that of pyruvate and lactate in the trained groups. Therefore, training *per se* seems to stimulate gluconeogenesis from amino acids.

Insulin chronically administered to the trained animals (TI) decreased glucose release without changing the other products, suggesting a prevalence of the indirect glycogen synthesis (thus decreasing the efflux of glucose), as discussed below. In contrast, insulin inhibition of gluconeogenesis seems to be dependent of extra-hepatic insulin actions, especially the inhibition of lipolysis in adipose tissue and proteolysis in muscle (37).

The study of liver metabolism with *in situ* perfusion, as it was conducted in this investigation, has the advantage of preserving the acinar functional structure of the liver and the periportal-perivenous sinusoidal flux (38). Therefore, in addition to training and insulin favoring gluconeogenesis and indirect glycogen synthesis, respectively, in the periportal zone, as a powerful activator of glycolysis in the perivenous zone insulin could be adding another mechanism to the reduced glucose release and increased glycogen content observed in group TI.

Insulin is a well-established promoter of muscle hyperplasia and hypertrophy, especially during (but not limited to) mammalian growth (39). The use of insulin by body builders as a synergistic agent of muscle hypertrophy, as well as some of its dangerous outcomes, are occasionally mentioned (5,40). The most common and potentially fatal of these is hypoglycemia, caused by insulin actions on glucose uptake and storage.

This work was a first approach to liver glucose metabolism in healthy strength-trained mice treated with low-dose regular insulin. Two combinations of gluconeogenic precursors – glycerol+lactate and alanine+glutamine – were employed, the second one being dependent on liver nitrogen disposal by transamination or ureagenesis. As these precursors enter the gluconeogenic pathway at different points, their separate infusion might reveal the sites most influenced by training and/or insulin in this model.

Resistance training had very marked effects on muscle strength, body weight, and adiposity. Insulin administration had no additional effect on these variables, and in fact reversed some of them on adipose tissue and liver metabolism promoted by resistance training, in addition to increasing the number of deaths in the group of trained animals that were given insulin. From the perspective of these results and the importance of liver metabolism for energy homeostasis, insulin administration is not additive to training in improving liver glucose metabolism. Additionally, this raises an alert for metabolically decompensated individuals, such as those who are obese and/or insulin resistant.
